# Does the Metabolically Healthy Obese Phenotype Protect Adults with Class III Obesity from Biochemical Alterations Related to Bone Metabolism?

**DOI:** 10.3390/nu11092125

**Published:** 2019-09-06

**Authors:** Ligiane Marques Loureiro, Suzane Lessa, Rodrigo Mendes, Sílvia Pereira, Carlos José Saboya, Andrea Ramalho

**Affiliations:** 1 Postgraduate Program, Nutritional Sciences, Federal University of Rio de Janeiro (UFRJ), Rio de Janeiro 21.941-902, Brazil; 2 Center for Research on Micronutrients (NPqM), Institute of NutritionJosué de Castro of UFRJ, Rio de Janeiro 21.941-902, Brazil; 3 Postgraduate Program, Applied Mathematics, Pontifical Catholic University of Rio de Janeiro, Rio de Janeiro 22.451-900, Brazil; 4 Multidisciplinary Center for Bariatric and Metabolic Surgery, Rio de Janeiro 22.280-020, Brazil; 5 Department of Social and Applied Nutrition of the Institute of Nutrition, UFRJ, Rio de Janeiro 21.941-902, Brazil

**Keywords:** metabolically healthy phenotype, obesity, adult, bone metabolism, micronutrients

## Abstract

Obesity negatively affects the relationship between markers and micronutrients of bone metabolism. Testing the hypothesis that the metabolically healthy obese phenotype might be protected by those alterations was the aim of this study. A cross-sectional study was carried out in adults with class III obesity classified in Metabolically Healthy Obese (MHO) and Metabolically Unhealthy Obese (MUHO), according to the National Cholesterol Education Program Expert Panel on Detection, Evaluation, and Treatment of High Blood Cholesterol in Adults (NCEP ATP III) criteria. Anthropometric, biochemical, and clinical variables were analyzed for sample characterization. To evaluate bone metabolism, markers (alkaline phosphatase and parathyroid hormone—PTH) and related nutrients (vitamin D, vitamin B12, calcium, phosphorus, magnesium, potassium and zinc) were analyzed. A total of 223 adults with class III obesity aged 41.20 ± 10.15 years were included. The MHO phenotype was identified in 32.73% of the sample. After logistic regression, it was observed that inadequacies of calcium (OR: 4.11; 95% CI: 2.33–6.66), phosphorus (OR: 3.03; 95% CI: 1.98–5.79), vitamin D (OR: 5.01; 95% CI: 2.92–6.71) and PTH (OR: 5.45; 95% CI: 4.49–6.74) were significantly higher in the MUHO group compared to the MHO Group. This study showed that the MHO phenotype does not protect adults from alterations in markers and micronutrients of bone metabolism. However, the MUHO phenotype presents a higher risk for alterations related to bone metabolism, which can favor the emergence of metabolic bone diseases.

## 1. Introduction

The increasing prevalence of obesity in adults worldwide has mainly occurred in class III obesity group (body mass index—BMI, ≥40 kg/m^2^), showing a rate significantly higher than those observed in other obesity categories [1]. Recently, evidence has suggested the existence of a link between the pathophysiological mechanisms of obesity and those of other syndromes mainly related to the compromise of bone [2,3] and liver metabolism [4].

Obesity is related to the highest prevalence of diseases such as the metabolic bone [2,3], which are characterized by alterations in some biochemical markers [5,6], favored by the inflammatory profile of obesity [7], besides the hormonal, enzymatic, and metabolic [8,9] alterations to which these individuals are more exposed.

Although obesity is a risk factor for a series of metabolic complications, the presence of the phenotype known as Metabolically Healthy (MH) has suggested that individuals that are classified as metabolically healthy obese (MHO) seem to be more protected from complications related to the severity of Non-Alcoholic Fatty Liver Disease (NAFLD), insulin resistance (IR), alterations in lipid profile and in inflammation markers, unlike those classified as Metabolically Unhealthy Obese (MUHO) [10,11]. The role of the MHO phenotype has been widely debated, although the lack of standardization for its classification plays an important role in the differences observed between the available studies that have reported controversial and conflicting results [12,13]. Different criteria for its identification have been adopted, and the most common criteria used in clinical practice and in epidemiologic researchers has been the Third Report of the National Cholesterol Education Program Expert Panel on Detection, Evaluation, and Treatment of High Blood Cholesterol in Adults (NCEP ATP III) [14], which considers factors such as MHO, the subject which shows alterations in <3 of the following 5 criteria used to diagnose metabolic syndrome (MS). More recently NAFLD, considered to be the manifestation of MS in the liver [15], has been investigated in these individuals due to its association with obesity. MHO individuals have lower liver fat accumulation when compared to MUHO individuals [10], which is related to increased risk factors for alterations in markers involved in the bone metabolism [16] of obese adults.

The literature shows that adults with class III obesity are more prone to have alterations in bone metabolism, which results in increased bone remodeling [5] in these individuals. Thus, it is important to evaluate the associations between metabolic alterations in obesity and serum concentrations of some markers, given their participation in essential metabolic and endocrine processes related to the maintenance of bone health [6]. However, there is a scarcity of reports in the literature that includes data relating aspects of bone metabolism to metabolic abnormalities found in phenotypes.

## 2. Materials and Methods

### 2.1. Participants

Adults of both genders were included, aged ≥20 and <60 years and body mass index (BMI) ≥40 kg/m^2^ (diagnosed with class III obesity). Exclusion criteria were: prior malabsorptive and restrictive surgeries, intestinal malabsorptive syndromes, neoplasia, use of drugs for weight loss, use of lipid lowering drugs, hypoglycemic drugs, alcohol intake exceeding 20 g/day for women and 40 g/day for men, use of multivitamin and mineral supplements, to be pregnant or a nursing mother, and presence of kidney failure and liver diseases, except NAFLD. This study was approved by the Research Ethics Committee of Hospital Universitário Clementino Fraga Filho (HUCFF) of the Universidade Federal do Rio de Janeiro (UFRJ) [Federal University of Rio de Janeiro] under the Scientific Advice n° 011/10 in accordance with Resolution n° 196 of the National Health Council and the Declaration of Helsinki. Inclusion of patients in the project was carried out through their formal authorization with the signing of the Informed Consent Form in two copies that were printed and delivered to each patient on the day of data collection.

### 2.2. Study Design

This is a descriptive, cross-sectional study with a convenience sample including adults with class III obesity [17], which attended the Multidisciplinary Center for Bariatric and Metabolic Surgery in Rio de Janeiro—RJ, Brazil. The sample was gathered using consultation of medical records. Data collection occurred from November 2014 to July 2016. The information collected included the patient identification, the drugs used, demographic data (such as gender, age, education, and other information), clinical evaluations, anthropometric, and biochemical assessments as well as lifestyle information (such as physical activity and sun exposure habits). For classification of the metabolically healthy obese (MHO) phenotype, the criterion used was proposed by the Third Report of the National Cholesterol education Program Expert Panel on Detection, Evaluation, and Treatment of High Blood Cholesterol in Adults (NCEP ATPIII) [14]. The subjects who showed alterations in ≥3 of the following 5 criteria were considered to be metabolically unhealthy obese (MUHO): (1) waist circumference (WC) >102 cm for men and >88 cm for women; (2) fasting glucose ≥100 mg/dL; (3) fasting triglycerides ≥150 mg/dL; (4) High Density Lipoprotein (HDL) <40 mg/dL for men and <50 mg/dL for women; and (5) Blood Pressure ≥130/≥85 mmHg.

### 2.3. Measurements

Anthropometric, clinical, and biochemical parameters were evaluated to characterize the sample and analyze the association among the markers of bone metabolism (regardless they are nutrients or not) with metabolic and bodily variables, according to MHO and MUHO classification. The anthropometric evaluation included the measurement of weight (using a Welmy^®^ electronic platform scale with maximum weighing capacity of 300 kg, São Paulo, Brazil) and height (using a Sany^®^ stadiometer, São Paulo, Brazil) for BMI calculation according to the World Health Organization (WHO) [18]; waist circumference (WC) was measured based on Lohman et al. [19] with cutoff points according to the NCEP ATP III [14], and the visceral adiposity index (VAI) was calculated according to Amato et al. [20]. All measurements were performed in duplicate by a single trained observer, variations up to 0.5 cm were accepted, and the mean was calculated. Physical activity was evaluated through the International Physical Activity Questionnaire (IPAQ) in its short version [21] and volunteers were classified into sedentary, inadequately inactive, active, or very active.

The clinical variables considered by the study were those determined from the frequencies of NAFLD and systemic hypertension (SH). Data on NAFLD and SH were obtained through the medical records of the patients. The diagnosis of NAFLD was determined by a complete abdominal ultrasound, conducted by a single physician with expertise on imaging diagnosis, following the methodology proposed by Pratt & Kaplan [22] using a unit C display, Philips^®^ 2–5 MHz Convex transducer (Barueri, São Paulo, Brazil). SH was diagnosed by a professional expert, following the methodology proposed by the VII Brazilian Guidelines of Systemic Hypertension (SOCIEDADE BRASILEIRA DE CARDIOLOGIA—SBC, 2016) [23] and with a ≥130/≥85 mmHg cutoff point in line with the NCEP-ATP III [14].

A total of 5 mL blood samples was obtained via venipuncture after 12 h of fasting for biochemical evaluations to determine glucose and basal insulin, lipid profile, inflammatory profile, and markers related to bone metabolism. The overall analyses were performed in laboratory with certifications, in partnership with the Center for Research in Micronutrients of the Institute of Nutrition of the UFRJ and the Multidisciplinary Center for Metabolic and Bariatric Surgery. Blood glucose was obtained by the enzymatic colorimetric method. The basal insulin was quantified by high performance liquid chromatography of reverse phase (RP-HPLC) and the cutoff point adopted was 24.9 IU/mL. Insulin resistance was estimated by the formula of the homeostasis model assessment of insulin resistance (HOMA-IR), with ≥2.5 cutoff point [24]. The serum concentrations of total cholesterol and triglycerides were analyzed by the enzymatic colorimetric method, and the Low-Density Lipoprotein Cholesterol (LDL-c) and High-Density Lipoprotein Cholesterol (HDL-c) fractions were obtained by the selective inhibition method. The cutoff point for total cholesterol and fractions, triglycerides, and fasting glucose were those established by the NCEP-ATP III criteria [15]. C-reactive protein (CRP) was quantified by the nephelometric method and the cutoff point to determine inflammation was >0.3 mg/dL [25].

Parathyroid hormone (PTH) was analyzed by the immunoenzymatic and chemiluminescence methods [26] and serum alkaline phosphatase (AP) by the kinetic colorimetric enzymatic method [27], both with cutting points for inadequacy >53.0 pg/mL (secondary hyperparathyroidism) and <130.0 U/L, respectively. Serum vitamins were quantified by the high-performance liquid chromatography-ultraviolet detection method (HPLC-UV) (Labtest Diagnóstica S.A., Lagoa Santa, Minas Gerais, Brazil). Vitamin B12 cutoff point was <271 pg/mL [28]. The nutritional status of vitamin D was analyzed by the quantification of the serum concentrations of 25(OH)D, and the cutoff points were ≤20 ng/mL (deficiency), ≥20 ng/mL and <29 ng/mL (insufficiency), and ≥30 ng/mL and <100 ng/mL (adequacy) [29]. For complementing vitamin D evaluation, a study on the sun exposure of the participants was carried out by the application of a protocol validated by Hanwell et al. [30]. Phosphorus, magnesium, potassium, and zinc were the serum minerals quantified by the calorimetric method, with the cutoff points respectively for inadequacy <2.5 mg/dL [31], <1.7 mg/dL [32], <2.7 mEq/dL [33], and <70 mcg/dL [34]. The nutritional status of calcium was determined by quantification of serum concentrations of ionic calcium, by direct dosage and by selective electrode. The cutoff point for inadequacy was <4 mg/dL [33].

### 2.4. Statistical Analysis

Statistical analyses were performed using the Statistical Package for the Social Sciences (SPSS) package for Windows version 21.0, IBM Corporation (Chicago, IL, USA). To verify the normality of the sample, the Kolmogorov-Smirnoff test was performed. Continuous variables were expressed as mean and standard deviation. Clinical, biochemical, anthropometric parameters and markers of bone metabolism according to the MHO and MUHO phenotypes were compared using the Student’s t-test. The Pearson’s chi-square test was applied to assess the categorical variables according to MHO and MUHO groups. Pearson’s chi-square test was used to test the homogeneity of proportions among the categorical variables, and Pearson’s Linear Correlation was used for the continuous variables. Multiple logistic regression analysis was performed to determine the odds ratio (OR) of markers of bone metabolism altered by the MHO and MUHO phenotypes. The significance level adopted was 5% (*p* < 0.05).

## 3. Results

### 3.1. General Characterization of the Studied Population

**Figure 1:**
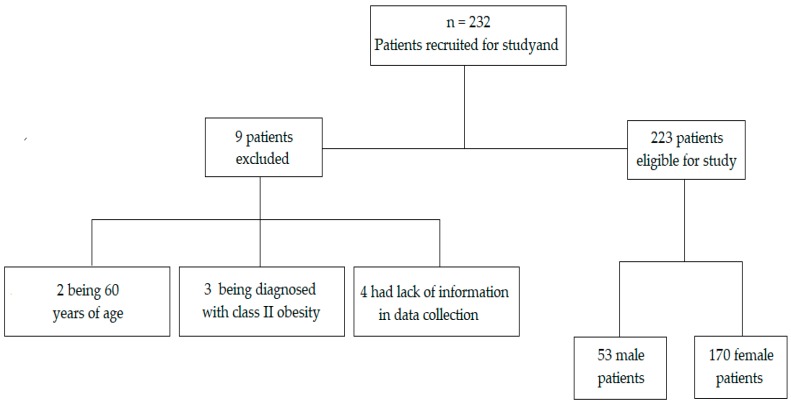
Flow chart of the study.

A total of 232 adults with obesity were recruited for study and were in the preoperative period of bariatric surgery. From these, a total of 9 adults were excluded from the study because they did not meet any of the eligibility criteria, two of them because they were 60 years of age, 3 because they were diagnosed with class II obesity and the remainder because they had important information which could not be answered at the time of the consultation with the nutritionist for data collection (Figure 1). Thus, the sample comprised 223 adults with class III obesity, mostly sedentary, 79.82%, with 76.23% females and 23.77% males, mean age of 41.20 ± 10.15 years, and no statistical difference between genders was observed. NAFLD was present in 97.58% of the studied sample.

**Figure 2:**
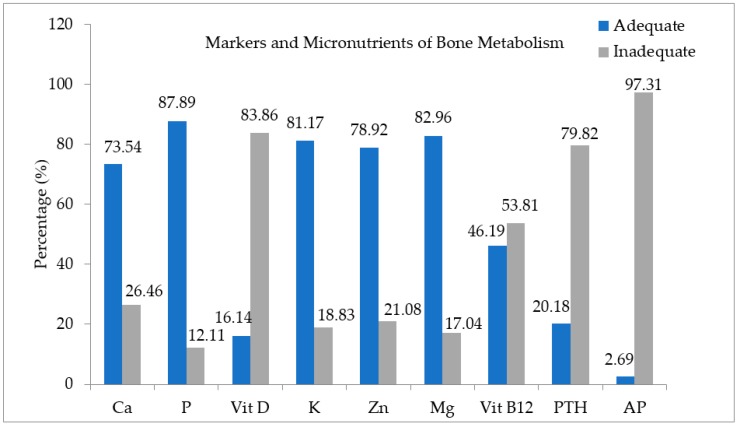
(%) of adequacy and inadequacy of markers and micronutrients of bone metabolism of the sample comprising 223 adults with obesity. Abbreviations: Ca: calcium; P: phosphorus; Vit. D: vitamin D; K: potassium; Zn: zinc; Mg: magnesium; Vit. B12: vitamin B12; PTH: Parathyroid hormone; AP: Alkaline Phosphatase.

With respect to the proportion of subjects in the study, 97.31% showed inadequacy of AP, 83.86% vitamin D deficiency, 79.82% inadequacy of PTH, 53.81% vitamin B12 deficiency, and 26.46% calcium deficiency (Figure 2). Sun exposure time (min) among the individuals with obesity was 13.2 ± 5.2 min/day, and no statistical difference was observed between them after their categorization into MHO and MUHO phenotypes.

### 3.2. Characterization of the Population According to the MHO and MUHO Phenotypes

Based on the NCEP-ATPIII criterion, the classification of subjects showed that 32.73% (73 out of 223) were categorized as MHO and 67.26% (150 out of 223) were categorized as MUHO. The mean age between the MHO and MUHO groups was 38.86 ± 10.65 and 42.34 ± 9.73 years (*p* = 0.020), respectively, and a statistical difference was found between phenotypes (*p* = 0.068).

Regarding the clinical, biochemical, and anthropometric parameters of the MHO and MUHO groups (Table 1), statistically significant differences were found between the means of VAI 4.28 ± 3.56 and 7.93 ± 8.70 (*p* = 0.001), of insulin (mg/dL) 16.36 ± 8.97 and 19.73 ± 11.67 (*p* = 0.018) and HOMA-IR 3.74 ± 2.32 and 5.40 ± 4.22 (*p* = 0.002) of the WHO and MUHO groups, respectively.

As for the other metabolic variables (clinical and biochemical) present in the NCEP-ATP III classification criteria, the prevalence of 36% of SH in the MHO group was significantly lower (*p* < 0.001) when compared to the MUHO group, where it reached 87%. Similarly, significantly lower values of fasting glucose (mg/dL) 91.03 ± 15.99 vs. 106.81 ± 30.42 (*p* < 0.001) and TG (mg/dL) 123.04 ± 140.94 vs. 190.25 ± 239.38 (*p* = 0.009) were found, in addition to significantly higher concentrations of HDL-c (mg/dL) 52.25 ± 11.64 vs. 42.49 ± 8.96 *(p* < 0.001) in the MHO group, when compared to the MUHO group. We observed that the prevalence of NAFLD was significantly higher (*p* < 0.001) in the MUHO group, when compared to the MHO (Table 1). In the analyzed profile of markers and micronutrients of bone metabolism selected for the study, AP showed a significant difference between the groups (*p* = 0.016), and was lower in the MHO group (Table 2).

### 3.3. Association and Odds Ratio (OR) of the Markers of Bone Metabolism, According to the MHO and MUHO Phenotypes

We observed a statistically significant difference (*p* = 0.031) in relation to the inadequacy of PTH between the groups that was lower in the MHO group, 12% (*n* = 9), when compared to the MUHO group, 24% (*n* = 38). No association between the phenotypes was found with the other evaluated markers (Table 3).

To investigate the risk factors for alteration in bone metabolism according to phenotypes, a logistical regression analysis was performed, which was adjusted for confounding variables (age, gender, physical activity and NAFLD). The MUHO phenotype was more associated with risk factors for alteration in markers and micronutrients of bone metabolism. This association remained even after adjusting clinical and biochemical variables and pathological diagnosis, the OR of the MUHO group for inadequacy of calcium (OR: 4.11; 95% CI: 2.33–6.66), phosphorus (OR: 3.03; 95% CI: 1.98–5.79) vitamin D (OR: 5.01; 95% CI: 2.92–6.71) and PTH (OR: 5.45, 95% CI: 4.49–6.74) were significantly higher compared to the MHO group (Table 4).

## 4. Discussion

This study presents two main findings. First, individuals with obesity, regardless of their classification into MHO or MUHO phenotypes, showed associations with alterations in the investigated markers and micronutrients of bone metabolism. Secondly, the combination of obesity with the metabolically unhealthy status has raised the risk for alterations in these markers and micronutrients, which are important for bone metabolism. The strong point in our study that it have been the first to report the relationship between the MH phenotype and a set of biochemical variables that are important for bone health in individuals with class III obesity, since the previous publications in the literature have evaluated classes I or II obesity and correlated the MH phenotype with diabetes mellitus [34], SH [35], NAFLD [10,36], cardiovascular diseases [37,38,39], and with a lower risk of mortality [40]. However, we assume as one of our limitations the absence of some variables (bone mineral density, vitamin K and osteocalcin, for example) related to the bone-metabolic and bone-muscle profile of the investigated sample. Even so, we believe that the set of biochemical markers (nutrients or not) selected here for study was able to demonstrate how relevant they are and how important the role they play is in the mechanisms related to the maintenance of bone health and prevention of various diseases, especially those of a bone-metabolic and muscular nature, as has been pointed out by several epidemiological studies cited here.

### 4.1. General Characterization of the Population, According to the MHO and MUHO Phenotypes

The prevalence of the MHO phenotype found in our study was 32.73%. Evidence has shown that 20%–30% of obese individuals are classified as MHO [41]. Other authors have described frequencies of 14.9% [42], 19.5% [35] and 36.6% [43]. Nonetheless, in these studies, different classes of obesity were evaluated using distinct methods for the classification of the MH phenotype, which could also justify the differences found.

Although both phenotypes showed significant inadequacy in four of the five variables comprising the NCEP-ATP III classification criterion (Table 1) except for AC, which we believe has not been deferred since the study comprises individuals with class III obesity [44] (Table 1). SH was the variable most strongly associated with the MH phenotype, with a high prevalence (87%) among the MUHO group. Our results corroborate previous findings [35,41,45]. Current evidence [36,45] also suggests that there are additional clinical characteristics of MHO that include the importance of lipid, hepatic, and inflammatory profiles in relation to the MH phenotype.

We found a high frequency para (97.58%) of NAFLD, mainly in the MUHO group (94%), which suggests the existence of an important relationship with the MH phenotype, particularly if considering that liver fat content as a marker that has been highlighted in the context of the definition of this phenotype. However, it was not possible to further investigate these results, since the diagnosis of NAFLD was carried out by abdominal US with no possibility of the evaluation of its severity.

Individuals with obesity present different patterns of body fat distribution related to distinct metabolic phenotypes [46]. In our study we found significantly smaller values of VAI in the MHO group, when compared to the MUHO. This finding suggests a difference between the MHO and MUHO phenotypes in the site of the largest body fat deposit.

In addition, the other differences in the clinical and metabolic profile observed in our study, such as the one verified in relation to the HOMA-IR parameter (Table 1), can be justified through several mechanisms that have not yet clarified for interaction between genetic, environmental, and behavioral factors.

### 4.2. Characterization of Biochemical Markers of Bone Metabolism, According to the MHO and MUHO Phenotypes

When we compared the profile of markers of bone metabolism, we observed that AP was related to the MH phenotype. Higher plasma concentrations of the marker were observed in the most compromised metabolic phenotype, MUHO (Table 2). AP is a marker of bone formation. When it is present at elevated levels on the surface of the cytoplasmic membranes of osteoblasts and pre-osteoblasts, AP contributes to the beginning of mineralization and the progressive growth of hydroxyapatite crystals [47]. In individuals with class III obesity, this alteration is normal [48]. This marker tends to be in greater concentration when there is vitamin D deficiency. Besides, it is still influenced by the homeostasis of PTH, calcium, and vitamin D [49,50]. Alterations in this triad of markers and micronutrients have been associated with unfavorable metabolic outcomes in relation to bone [51,52] and hepatic health [44,53]. Therefore, even though the other markers did not differ in the MHO and MUHO phenotypes, we believe it is important to further evaluate the significance of the observed alterations, as they are markers of increased bone remodeling.

### 4.3. Association and Odds Ratio of Markers and Micronutrients of Bone Metabolism, According to the MHO and MUHO Phenotypes

Independently of the MHO and MUHO phenotypes, we observed high frequencies of inadequacy, mainly of AP and PTH markers, in addition to vitamin D, vitamin B12 and calcium (Figure 1). Obesity per se can alter the production pattern of biochemical markers of bone metabolism, regardless of whether the markers are nutrients or not, as they depict bone formation or reabsorption [54,55]. Such inadequacies often occur in individuals with class III obesity [51,56,57]. They are related to pathogenesis of various diseases, including bone-metabolic diseases and bone-muscle diseases [52,58] and NAFLD [53].

Considering the high prevalence of NAFLD in our subjects, mainly in the MUHO group (94%), we assume it may be related to alterations in some of these evaluated markers, especially vitamin D deficiency [44,59], since the first hydroxylation of this vitamin occurs in the liver and when this organ is functionally compromised it can interfere in the metabolism of vitamin D, and consequently, in its bioavailability. Regarding micronutrient deficiencies, our findings are also in line with previous studies [60,61] that have pointed out that vitamins (vitamin D and vitamin B12) and minerals (calcium, phosphorus, potassium, zinc, and magnesium) are often inadequate in adults with obesity. We also observed varying degrees of deficiency in all these micronutrients, although no significant difference was found according to the MHO and MUHO phenotypes (Table 3). However, after logistic regression and adjustments in confounding factors, the MUHO phenotype presented high and significant values of OR for calcium (OR: 4.11 *; 95% CI: 2.33 *—6.66), phosphorus (OR: 3.03; 95% CI: 1.98–5.79) and vitamin D (OR: 5.01 *; 95% CI: 2.92–6.71) that have been associated with the greatest risk for alteration in bone metabolism (Table 4).

Several studies indicate that deficiencies of calcium, vitamin D and phosphorus in the long term, especially when associated with obesity as we observed in our study, increase the risk of fractures and can also be associated with the greater prevalence of osteoporosis, osteoarthritis, sarcopenia [51,52,62], in addition to the osteosarcopenic obesity [58,63]. After adjusting for age, gender, physical activity, and the presence of NAFLD, we observed an association between calcium, vitamin D and phosphorus in the MUHO phenotype, thus showing that such variables appear to have been influenced by the most compromised metabolic profile. These findings are of great relevance since vitamin D is necessary for maintaining normal levels of calcium and phosphorus, which in turn are necessary for proper bone mineralization, among many other functions [64]. Its deficiency was related to loss of bone mass in individuals with obesity [65]. Vitamin D deficiency causes increased bone turnover, with an increase in markers such as alkaline phosphatase, as we observed in our study, and it is associated with an increase in fracture risk [51].

Inadequacy of PTH was the only variable associated with the MH phenotype (Table 3). We found significantly higher inadequacy of this hormone in the MUHO group, with a 24% prevalence of secondary hyperparathyroidism. Besides, this group showed significantly greater risk for alteration in PTH (OR: 5.45; 95% CI: 4.49–6.74) when compared to the MHO group (OR: 5.11; 95% CI: 4.12–6.48), thus reinforcing the relationship with the MH phenotype (Table 4). Secondary hyperparathyroidism is very frequent mainly in individuals with class III obesity and it interacts with other nutritional deficiencies [57,66], as we found in our study. Vitamin D and calcium deficiencies and hormonal alterations such as secondary hyperparathyroidism when untreated result in bone diseases [48,67]. Based on our findings, we postulate that obesity *per se* already promotes detrimental alterations in markers of bone metabolism independently of the MH phenotype. Our findings also emphasize the importance of considering this phenotype, especially when it is associated with class III obesity, in order to subsidize nutritional interventions in clinical practice, considering the differences observed between the metabolic profiles of the MHO and MUHO phenotypes that were studied.

## 5. Conclusions

The Metabolically Healthy Obesity Phenotype does not protect adults with class III obesity from developing biochemical alterations related to bone metabolism. Nonetheless, this class of obesity, when associated with the Metabolically Unhealthy Phenotype, presented a higher risk of alterations to PTH, Calcium, Vitamin D, and Phosphorus, thus favoring the emergence of metabolic bone diseases. We suggest conducting new studies, preferably with follow-ups, to deeply investigate the significance of our findings and point out the possible trigger factors for the alterations encountered and their actual impact on the bone health of adults with obesity. In this way, strategies in clinical practice can be adopted in order to minimize undesirable effects on this population.

## Figures and Tables

**Table 1:** Clinical, biochemical and anthropometric characteristics of the metabolically healthy obese (MHO) and metabolically unhealthy obese (MUHO) phenotypes.

General Characteristics		MHO (*n* = 73) Mean ± SD	MUHO (*n* = 150) Mean ± SD	*p*-Value
**Clinical Variables**				
Systolic H (mmHg)		123.3 ± 23.4	140.0 ± 30.4	0.001 *
Diastolic H (mmHg)		76.6 ± 18.0	90.2 ± 24.4	0.001 *
SH (%)	Yes	35.6 (*n* = 26)	87.3 (*n* = 131)	0.001 **
	No	64.4 (*n* = 47)	12.7 (*n* = 19)	
NAFLD (%)	Yes	83.6 (*n* = 61)	94.0 (*n* = 141)	0.025 **
	No	16.4 (*n* = 12)	6.0 (*n* = 9)	
**Anthropometric Variables**				
Weight (Kg)		118.4 ± 16.3	118.0 ± 19.8	0.876
Height (meters)		1.6 ± 0.1	1.6 ± 0.1	0.723
BMI (Kg/m^2^)		42.9 ± 4.4	42.5 ± 4.8	0.465
AC (cm)		118.1 ± 11.7	120.5 ± 13.8	0.186
VAI		4.2 ± 3.5	7.9 ± 8.7	0.001 *
**Biochemical Variables**				
Glucose (mg/dL)		91.3 ± 15.9	106.8 ± 30.4	0.001 *
Insulin (mg/dL)		16.3 ± 8.9	19.7 ± 11.6	0.018 *
HOMA-IR		3.7 ± 2.3	5.4 ± 4.2	0.002 *
Total Cholesterol (mg/dL)		197.6 ± 36.1	199.0 ± 57.1	0.826
LDL-c (mg/dL)		121.1 ± 31.6	120.3 ± 35.5	0.867
HDL-c (mg/dL)		52.2 ± 11.6	42.4 ± 8.9	<0.001 *
Triglycerides (mg/dL)		123.0 ± 140.9	190.2 ± 239.3	0.009 *
CRP (mg/dL)		0.8 ± 0.7	0.9 ± 0.9	0.427

* *t*-Student test (*p* < 0.05); ** Pearson’s Chi-Square (X^2^) Test (*p* < 0.05); Abbreviations: SD: standard deviation; H: Hypertension; SH: Systemic Hypertension; NAFLD: Non-Alcoholic Fatty Liver Disease; BMI: Body Mass Index; AC: Abdominal Circumference; VAI: Visceral Adiposity Index; HOMA-IR: Homeostasis Model Assessment—Insulin Resistance; LDLc: Low Density Lipoprotein Cholesterol; HDL-c: High Density Lipoprotein Cholesterol; TG: Triglycerides; CRP: C-Reactive Protein.

**Table 2:** Profile of biomarkers and micronutrients of bone metabolism according to the MHO and MUHO phenotypes.

Variables	MHO (*n* = 73)	MUHO (*n* = 150)	*p*-Value
	Mean/SD	Mean/SD	
Calcium (mg/dL)	3.9 ± 1.4	3.9 ± 1.8	0.987
Phosphorus (mg/dL)	3.5 ± 0.5	3.6 ± 0.6	0.799
25(OH)D (ng/mL)	22.3 ± 7.7	22.6 ± 8.3	0.747
Potassium (mg/dL)	5.2 ± 6.9	4.6 ± 4.8	0.527
Zinc (mg/dL)	99.9 ± 32.3	99.9 ± 27.2	0.994
Magnesium (mg/dL)	2.0 ± 0.1	2.0 ± 0.2	0.168
Vitamin B12 (pg/mL)	370.5 ± 200.5	344.1 ± 183.4	0.345
PTH (pg/mL)	42.0 ± 18.1	42.7 ± 16.0	0.779
AP (U/L)	70.5 ± 27.5	80.1 ± 27.5	0.016 *

* *t*-Student test (*p* < 0.05). Abbreviations: SD: Standard Deviation; PTH: Parathyroid hormone; AP: Alkaline Phosphatase.

**Table 3:** Association between markers and micronutrients of bone metabolism according to the MHO and MUHO phenotypes.

Variables	Status Nutritional	MHO (*n* = 73)	MUHO (*n* = 150)	*p*-Value
Calcium (%)	Adequate	79.5 (*n* = 58)	70.7 (*n* = 106)	0.108
	Inadequate	20.5 (*n* = 15)	29.3 (*n* = 44)	
Phosphorus (%)	Adequate	89.0 (*n* = 65)	87.3 (*n* = 131)	0.449
	Inadequate	11.0 (*n* = 8)	12.7 (*n* = 19)	
25(OH)D (%)	Deficient	38.4 (*n* = 28)	39.3 (*n* = 59)	0.720
	Insufficient	47.9 (*n* = 35)	43.3 (*n* = 65)	
	Sufficient	13.7 (*n* = 10)	17.4 (*n* = 26)	
Potassium (%)	Adequate	84.9 (*n* = 62)	79.3 (*n* = 119)	0.207
	Inadequate	15.1 (*n* = 11)	20.7 (*n* = 31)	
Zinc (%)	Adequate	75.3 (*n* = 55)	80.7 (*n* = 121)	0.228
	Inadequate	24.7 (*n* = 18)	19.3 (*n* = 29)	
Magnesium (%)	Adequate	89.0 (*n* = 65)	80.0 (*n* = 120)	0.064
	Inadequate	11.0 (*n* = 8)	20.0 (*n* = 30)	
Vitamin B12 (%)	Adequate	42.5 (*n* = 31)	48.0 (*n* = 72)	0.263
	Inadequate	57.5 (*n* = 42)	52.0 (*n* = 78)	
PTH (%)	Yes	12.3 (*n* = 9)	24.0 (*n* = 36)	0.029 *
	No	87.7 (*n* = 64)	76.0 (*n* = 114)	
AP (%)	Altered	2.7 (*n* = 2)	2.7 (*n* = 4)	0.640
	Unaltered	97.3 (*n* = 71)	97.3 (*n* = 146)	

* Pearson’s Chi-Square (X^2^) Test (*p* < 0.05). 25(OH)D: Vitamin D; PTH: Parathyroid Hormone (Yes or No for Hyper-Parathyroidism); AP: Alkaline Phosphatase.

**Table 4:** Risk factors for alteration in bone metabolism according to the MHO and MUHO phenotypes.

Variables	MHO (*n* = 73)	MUHO (*n* = 150)
	AOR	AOR
Calcium (mg/dL)	3.1	4.1 *
	(2.2–6.0)	(2.3–6.6)
Phosphorus (mg/dL)	2.0	3.0 *
	(0.8–4.6)	(1.9–5.7)
25(OH)D (ng/mL)	2.8	5.0 *
	(1.9–3.9)	(2.9–6.7)
Potassium (mg/dL)	1.1	1.7
	(0.4–2.8)	(0.6–3.0)
Zinc (mg/dL)	1.9	1.8
	(0.9–2.9)	(0.8–3.9)
Magnesium (mg/dL)	1.0	3.5
	(0.9–1.9)	(2.0–4.7)
Vitamin B12 (pg/mL)	0.8	2.5
	(1.1–3.4)	(1.5–5.4)
PTH (pg/mL)	5.1	5.4 *
	(4.1–6.4)	(4.4–6.7)
AP (U/L)	2.3	2.0
	(0.8–4.6)	(0.6–4.6)

AOR: Adjusted Odds Ratio (95% Confidence Interval) adjusted for age; gender; physical activity; hepatic steatosis. All variables had a significant association with * *p* < 0.05.
